# Higher order scaffoldin assembly in *Ruminococcus flavefaciens* cellulosome is coordinated by a discrete cohesin-dockerin interaction

**DOI:** 10.1038/s41598-018-25171-8

**Published:** 2018-05-03

**Authors:** Pedro Bule, Virgínia M. R. Pires, Victor D. Alves, Ana Luísa Carvalho, José A. M. Prates, Luís M. A. Ferreira, Steven P. Smith, Harry J. Gilbert, Ilit Noach, Edward A. Bayer, Shabir Najmudin, Carlos M. G. A. Fontes

**Affiliations:** 1 CIISA – Faculdade de Medicina Veterinária, ULisboa, Pólo Universitário do Alto da Ajuda, Avenida da Universidade Técnica, 1300-477 Lisboa, Portugal; 2 UCIBIO-REQUIMTE, Departamento de Química, Faculdade de Ciências e Tecnologia, Universidade Nova de Lisboa, 2829-516 Caparica, Portugal; 3 Department of Biomedical and Molecular Sciences, Queen’s University, Kingston, ON K7L 3N6 Canada; 4 Institute for Cell and Molecular Biosciences, Newcastle University, The Medical School, Newcastle upon Tyne, NE2 4HH United Kingdom; 5 Department of Biomolecular Sciences, The Weizmann Institute of Science, Rehovot, 76100 Israel; 6 NZYTech genes & enzymes, Estrada do Paço do Lumiar, 1649-038 Lisboa, Portugal

## Abstract

Cellulosomes are highly sophisticated molecular nanomachines that participate in the deconstruction of complex polysaccharides, notably cellulose and hemicellulose. Cellulosomal assembly is orchestrated by the interaction of enzyme-borne dockerin (Doc) modules to tandem cohesin (Coh) modules of a non-catalytic primary scaffoldin. In some cases, as exemplified by the cellulosome of the major cellulolytic ruminal bacterium *Ruminococcus flavefaciens*, primary scaffoldins bind to adaptor scaffoldins that further interact with the cell surface via anchoring scaffoldins, thereby increasing cellulosome complexity. Here we elucidate the structure of the unique Doc of *R. flavefaciens* FD-1 primary scaffoldin ScaA, bound to Coh 5 of the adaptor scaffoldin ScaB. The *Rf*CohScaB5-DocScaA complex has an elliptical architecture similar to previously described complexes from a variety of ecological niches. ScaA Doc presents a single-binding mode, analogous to that described for the other two Coh-Doc specificities required for cellulosome assembly in *R. flavefaciens*. The exclusive reliance on a single-mode of Coh recognition contrasts with the majority of cellulosomes from other bacterial species described to date, where Docs contain two similar Coh-binding interfaces promoting a dual-binding mode. The discrete Coh-Doc interactions observed in ruminal cellulosomes suggest an adaptation to the exquisite properties of the rumen environment.

## Introduction

The cellulosome is a highly intricate molecular nanomachine produced by anaerobic microorganisms to efficiently deconstruct complex plant cell wall polysaccharides, such as cellulose and hemicellulose. It consists of a multi-protein complex with several independent enzymatic components arranged around a molecular scaffold, termed scaffoldin. Cellulosomes combine an extensive cohort of enzymes, including glycoside hydrolases, pectate lyases and carbohydrate esterases. The assembly of different enzymes into the cellulosome is suggested to promote protein stability while potentiating the synergism of biocatalysts displaying complementary activities^1,2^. This process allows anaerobic microorganisms to gain a critical advantage when extracting energy in highly competitive ecological niches and is critical to the recycling of carbon between microbes, herbivores and plants. Furthermore, cellulases and hemicellulases have captured the attention of several biotechnology-based industries due to their potential application for the bio-conversion of plant biomass into simpler molecules of importance to the production of renewable fuels and presenting biomedical applications^3–7^. Protein-protein interactions established between dockerin (Doc) modules, located in the cellulosomal enzymes, and cohesin (Coh) modules of the scaffoldin molecule are the primary force for cellulosomal assembly.

**Figure 1:**
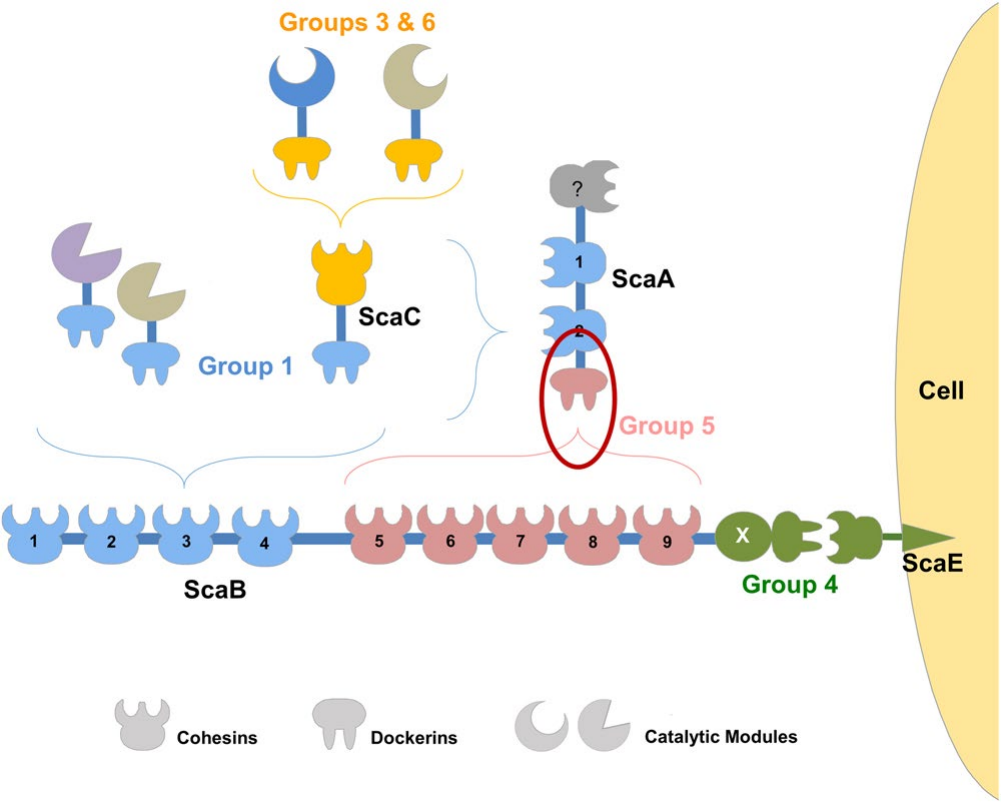
Cellulosome of *R. flavefaciens* strain FD-1 displaying the different group-specific Coh-Doc interactions involved in assembly of the multi-enzyme complex. The scheme is color-coded to highlight the four subgroups of cohesin-dockerin specificities: Dockerins and cognate cohesin counterparts of the different groups are marked in blue (Group 1 dockerins), yellow (Groups 3 and 6), green (Groups 2 and 4) and red (Group 5), respectively. Group 2 dockerins are truncated derivatives of group 4 and are not represented in the figure for simplification. The red oval marks the complex between DocScaA and CohScaB, representing the structure reported in this work.

Previously, extensive structural and biochemical characterization of type I and type II cohesin-dockerin (Coh-Doc) complexes revealed the molecular determinants of cellulosome assembly in *Clostridium thermocellum, Clostridium cellulolyticum, Pseudobacteroides cellulosolvens and Acetivibrio cellulolyticus*, species that colonize different ecological niches^8–11^. In general, Coh-Doc complexes involved in cellulosome assembly are classified as type I, while type II Coh-Doc interactions are involved in the attachment of cellulosomes onto the cell surface^12–14^, with the exception of *P. cellulosolvens*, wherein the Coh-Doc type interactions are reversed^11^ In contrast, the relevance of cellulosomes to fiber digestion in the rumen remains largely unexplored. The rumen, a large fermentation chamber located in the first portion of the gastrointestinal tract of ruminant mammals, is a highly dynamic and populated ecological niche colonized by a diverse microbiota that is highly effective in the deconstruction of recalcitrant carbohydrates^15^. To date *Ruminococcus flavefaciens*, a Gram-positive anaerobic bacterium of the Firmicutes phylum, is the only ruminal species that has been shown to assemble a definite cellulosome^16^. Intriguingly, various *R. flavefaciens* subspecies colonize the rumen, each displaying a different array of scaffoldins and dockerin-bearing proteins (enzymes) leading to different cellulosome architectures^17,18^. The proteome of *R. flavefaciens* strain FD-1 contains 223 dockerin-containing proteins (of which 154 are known carbohydrate-active enzymes)^18^, revealing the most diverse enzymatic system ever described in a cellulosome^19^ (Fig. 1). Based on primary sequence homology, *R. flavefaciens* Docs have been organized into six groups^20^. Recently, this classification was found to be functionally relevant^21^, with different Doc groups displaying different binding specificities. Thus, the 96 group 1 Docs of *R. flavefaciens* FD-1 bind to the two cohesins of primary scaffoldin ScaA and cohesins 1 to 4 of the adaptor scaffoldin ScaB. Hemicellulases primarily contain group 3 or 6 Docs that specifically bind to adaptor scaffoldin ScaC, whose group 1 Doc locks onto the Cohs of ScaA or Cohs 1–4 of ScaB^22,23^. Binding of the group 4 Doc of ScaB to the Coh of a cell surface protein, ScaE, revealed the mechanism used by *R. flavefaciens* strain FD-1 to anchor the entire cellulosome to the cell envelope. Not only cellulosomes are tethered to the cell surface but also single proteins as they contain Docs that bind specifically to cell surface Cohs rather than to cellulosomal Cohs. These Docs were classified into groups 4 and 2. Intriguingly, Group 2 Docs are truncated derivatives of group 4 Docs that retain the capacity to bind Cohs^21^. Finally, ScaA Doc, the unique member of group 5, binds exclusively to ScaB Cohs 5–9. This latter interaction has a central role in cellulosomal assembly as it allows the binding of up to five ScaA primary scaffoldins to ScaB and up to 10 more enzymes to a single cellulosome (Fig. 1).

Initial studies of *R. flavefaciens* Coh and Doc modules suggested that these sequences diverge at the primary sequence level from the previously described type I and type II modules and were, therefore, collectively classified as type III^23–26^. Until recently, only a single crystal structure of a type III Coh-Doc complex had been reported, comprising the X-module associated group 4 Doc of the cellulose-binding protein, CttA, bound to ScaE Coh. The structural divergence from previously described type I and II Coh-Doc complexes is pronounced, especially with respect to the Doc module. The CttA’s XDoc dyad has 5 *α*-helices instead of the typical three 3 *α*-helices and three inserts that act as structural buttresses to reinforce the stalk-like conformation of the X module^27^. Similar to CttA, ScaB possesses a conserved XDoc modular dyad at its C terminus. More recently three additional structures of *R. flavefaciens* strain FD-1 Coh-Doc complexes, namely ScaC Coh bound to a group 3 Doc^22^, a ScaA Coh bound to a group 1b Doc and a ScaB Coh bound to a group 1a Doc^28^, revealed the exquisite properties of rumen cellulosomes. While the first of the three is very similar to the previously described type I complexes, Coh-Doc complexes involving *R. flavefaciens* strain FD-1group 1 Docs do not bear much homology with any other complexes described to date. Although these three complexes are responsible for the integration of enzymes into the primary scaffoldins, either directly or through an adaptor scaffoldin, none of them possesses a dual-binding mode as observed in other cellulosomes^22,28^.

Here, we report the crystal structure of the *R. flavefaciens* strain FD-1 Coh-Doc complex established between ScaA Doc and the fifth cohesin of ScaB (*Rf*CohScaB-DocScaA). It is significant and revealing that ScaA Doc displays a unique sequence which exhibits an atypical Ca^2+^-binding site, due to several sequence alterations and a 12-residue insert in the midst of the second Ca^2+^-coordination loop. Comprehensive biochemical analysis of the CohScaB-DocScaA interaction, informed by the structural data, suggests an atypical single-binding mode. Thus, in contrast to the other known cellulosomes, this work supports the view that in *R. flavefaciens* cellulosome, protein assembly is the result of exclusively single-binding mode Coh-Doc interactions.

## Results and Discussion

Previous studies have shown that the Doc module of *R. flavefaciens* strain FD-1primary scaffoldin ScaA (*Rf*DocScaA) interacts exclusively with Cohs 5 to 9 of scaffoldin ScaB^21,24^. Intriguingly, *Rf*DocScaA is the lone member of the group 5 dockerins and therefore exhibits a unique sequence in strain FD-1. Moreover, the known orthologues in other *R. flavefaciens* strains each displays a similarly unique group 5-related sequence in the respective strain^20^. Thus, the CohScaB-DocScaA interaction is highly specific and central for *R. flavefaciens* cellulosome organization. Of the five possible *Rf*DocScaA-CohScaB complexes, the one involving the fifth ScaB cohesin (*Rf*CohScaB5) with *Rf*DocScaA displayed the highest levels of expression^21^. Here, the structure of *R. flavefaciens* strain FD-1 DocScaA in complex with the fifth cohesin from ScaB, for simplification designated *Rf*CohScaB5-DocScaA, was solved. Established *Escherichia coli* co-expression strategies for the production and purification of Coh-Doc complexes generated sufficient quantity of highly pure protein complexes to obtain good quality crystals.

### Structure of *Rf*CohScaB5-DocScaA protein complex

**Figure 2:**
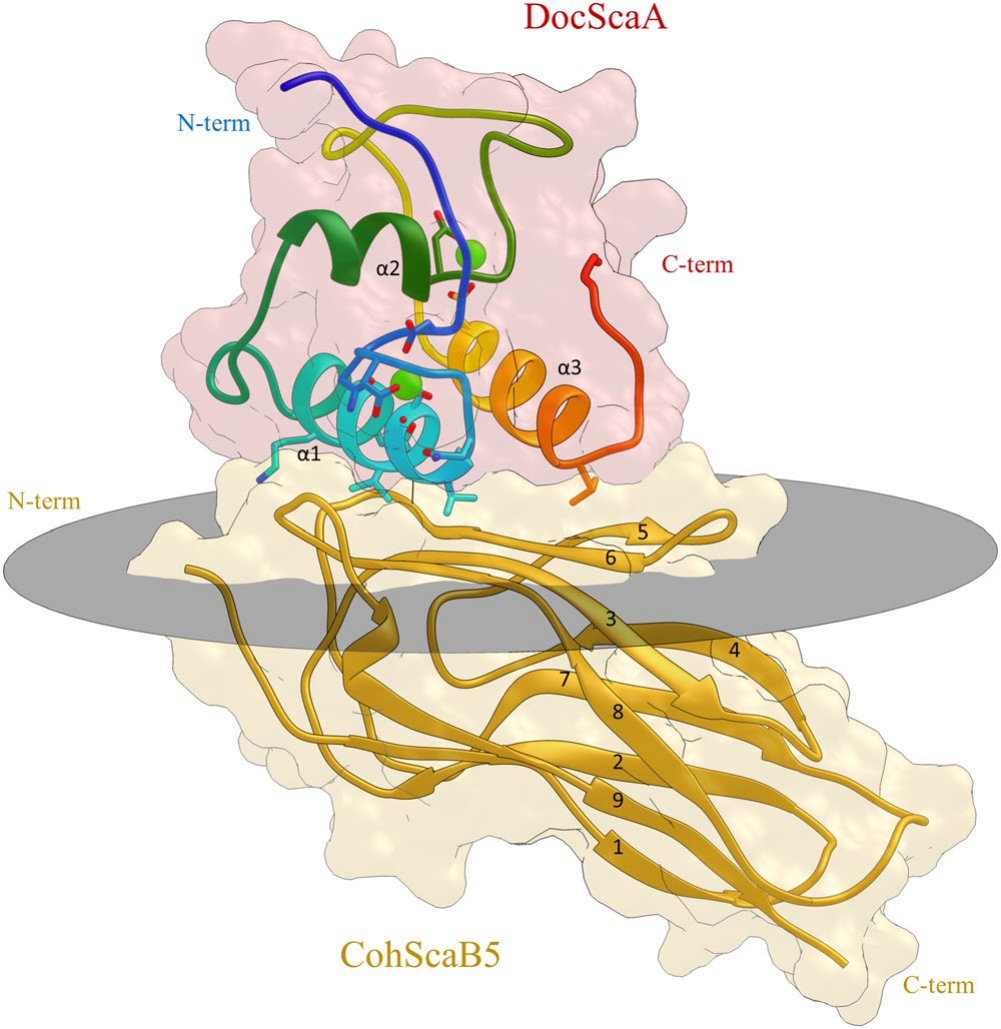
Structure of the *Rf*CohScaB5-DocScaA complex. Structure of the *Rf*CohScaB5-DocScaA complex with the dockerin in dark red and the cohesin in gold. The molecular surface of each module is represented in transparent colors. Under the transparent molecular surface and above the grey oval disk that marks the plane defined by the Coh 8–3–6–5 β-sheets, a ribbon representation shows the three Doc α-helices labeled α1, α2 and α3. Below the grey oval disk a ribbon representation of the cohesin shows each of the 9 β-strands, labeled from 1 to 9. Ca^2+^ ions are depicted as green spheres.

The *Rf*CohScaB5-DocScaA crystal structure was solved by molecular replacement (Fig. 2). The best crystals belonged to space group P2_1_ with unit cell dimensions of *a* = 30.1 Å, *b* = 142.9 Å, *c* = 46.6 Å, α = γ = 90 and β = 90.75. The *Rf*CohScaB5-DocScaA complex displayed an elongated comma shape with overall dimensions of 60 × 50 × 25 Å and included residues 740–877 from *Rf*CohScaB5 and 548–730 from *Rf*DocScaA. The structure included two molecules of the *Rf*CohScaB5-DocScaA heterodimer in the asymmetric unit, with each Doc coordinating two calcium (Ca^2+^) ions, as well as 1 acetonitrile and 225 water molecules. The crystallographic dimer resulted from interactions between two *Rf*CohScaB5 modules (chains A and C). Thus, chain A CohScaB5 O of Thr-743, Oγ of Ser-745 and Oδ1 of Asn-769 interact via hydrogen bonds with chain B CohScaB5 Oγ1 of Thr-752, N of Asp-869 and N of Leu-867, respectively. Thirty non-bonded contacts also contribute to the dimerization (not shown). The biological relevance of these crystallographic interactions, if any, is presently unclear. Data collection and structure refinement statistics are shown in Table 1.

**Table 1:** X-ray diffraction data collection and refinement statistics for *Rf*CohScaB5-DocScaA.

**Data collection**	
Beamline	PROXIMA-1, Soleil
Space Group	P12_1_1
Wavelength (Å)	0.82
**Unit-cell parameters**	
a, b c (Å)	30.09, 142.90, 46.59
α, β, γ (°)	90, 90.75, 90
V_M_^♯^ (Å^3^ Da^−1^)	1.89
Solvent Content (%)	35
Resolution limits (Å)	46.58–1.98 (2.07–1.98)
No. of observations	182195 (13279)
No. of unique observations	26476 (2602)
Multiplicity	6.9 (6.9)
Completeness (%)	99.9 (99.7)
<I/σ(I)>	8.85 (3.96)
CC_1/2_†	0.995 (0.974)
Wilson B-factor	22.71
R_merge_^‡^	0.098 (0.294)
R_p.i.m._^¥^	0.037 (0.085)
**Structure refinement**	
R-work^§^, R-free^¥^	0.182, 0.214
No. of non-H atoms	3519
Macromolecules	3287
Ligands	7
Water	225
Protein residues	449
RMS(bonds)	0.010
RMS(angles)	1.4
Ramachandran favored (%)	96
Ramachandran outliers (%)	0
Clash score	0.61
Average B-factor	33.80
macromolecules	33.80
ligands	39.30
solvent	33.50
PDB accession code	5N5P

Values in parenthesis are for the highest resolution shell.

^#^Matthews coefficient^50^.

^†^CC_1/2_ = the correlation between intensities from random half‐dataset (Diederichs & Karplus, 2013).

^‡^*R*_*merge*_ = *Σ*_*hkl*_ *Σ*_*i*_ *|I*_*i*_*(hkl)* − <*I(hkl)*>*|/Σ*_*hkl*_ *Σ*_*i*_ *I*_*i*_*(hkl)*, where *I*_*i*_*(hkl)* is the *i*th intensity measurement of reflection *hkl*, including symmetry-related reflections and <*I(hkl)*> is its average.

^¥^${R}_{p.i.m.}=\frac{{\sum }_{hkl}\sqrt{1/(n-1)}{\sum }_{i=1}^{n}|{I}_{i}(hkl)-I^{\prime} (hkl)|}{{\sum }_{hkl}{\sum }_{i=1}^{n}{I}_{i}(hkl)}$, a redundancy-independent version of R_merge_.

^*§*^R_work_ = *Σ*_*hkl*_*||F*_*obs*_*|* − *|F*_*calc*_*||/Σ*_*hkl*_*|F*_*obs*_*|*; ^¥^R_*free*_ as R_*work*_, but summed over a 5% test set of reflections.

### Structure of ScaB Coh5

*Rf*CohScaB5 displays an overall typical elliptical Coh structure containing nine *β*-strands forming two *β*-sheets in an elongated *β*-barrel displaying a classical “jelly-roll fold”. *β*-strands 9, 1, 2, 7, 4 comprise one sheet while *β*-strands 8, 3, 6, 5 are positioned on the opposite face. With the exception of *β*-strands 1 and 9, which align parallel to each other and close the jelly-roll, all remaining β-strands are antiparallel (Fig. 2). Notably, with the exception of a very poorly defined 3_10_-helix formed by residues Thr-862 to Lys-864, there are no structural motifs other than *β*-strands (Fig. 2). This observation contrasts with several bacterial Cohs where *β*-flaps are commonly found interrupting *β*-strand 4 or 8, including those from *Acetivibrio cellulolyticus* (PDB code 4UYP), *Pseudobacteroides cellulosolvens* (PDB code 1TYJ) or *R. flavefaciens* strain FD-1 ScaC Coh (PDB code 5LXV)^9,22,29^. The distinct *α*-helix commonly found between *β*-strands 4 and 5 in other Cohs is also absent. This particularity is shared with the recently described structures of *Rf*CohScaB3 (PDB code 5AOZ) and *Rf*CohScaA2 (PDB code 5M2S) which are the most homologous relevant *Rf*CohScaB5 structural homologs (with a Z-score of 8.1, rmsd of 1.78 Å and sequence identity of 27% over 127 aligned residues and Z-score of 7.9, rmsd of 1.76 Å and sequence identity of 23% over 127 aligned residues, respectively)^28^. Other structural homologs include the type I *Acetivibrio cellulolyticus* CohScaC3 (PDB code 4UYP) with a Z-score of 8.0, rmsd of 1.81 Å and 14% sequence identity over 125 aligned residues, and the type I *P. cellulosolvens* CohScaB7 (PDB code 4UMS), with a Z-score of 9.0, rmsd of 1.87 Å and sequence identity of 20% over 129 aligned residues.

### Structure of ScaA Doc

**Figure 3:**
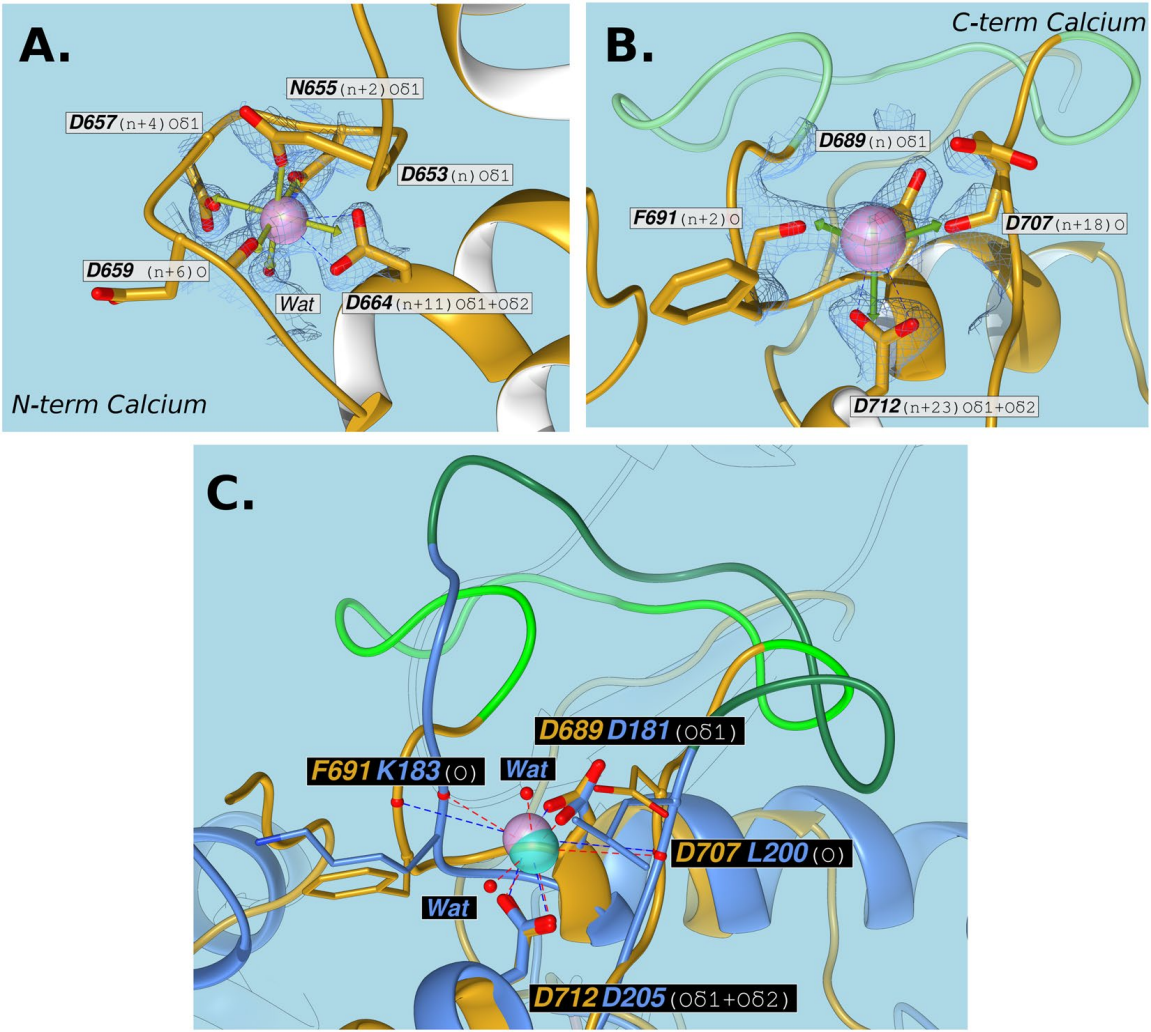
Calcium-coordination geometry at the N-terminal and C-terminal F-hand motifs of *R. flavefaciens* DocScaA. Panels A and B show a representation of *Rf*DocScaA N- and C-terminal Ca^2+^-coordination regions, respectively. In both panels the amino-acid residues involved in the metal coordination are depicted as sticks, surrounded by a mesh representation of the Refmac5 maximum-likelihood σ_A_–weighted 2F_o_−F_c_ electron density map contoured at 1σ (0.46 electrons/A^3^). The labels show the *Rf*DocScaA residue and coordination position numbers and also the atoms involved. Both calcium ions are depicted as purple spheres and are overlaid with an idealized geometry representation (green arrows), which is pentagonal bipyramidal for the N-terminal Ca^2+^ (Panel A) and tetrahedral for the C-terminal Ca^2+^ (Panel B). A single water molecule (Wat) completes the coordination sphere of the N-terminal Ca^2+^ ion (Panel A). The bidentate nature of the Asp-664 and Asp-712 coordination is highlighted with blue dashed lines (Panels A and B). The 12-residue insert at the C-terminal calcium coordination loop is colored in light green (Panel B). Panel C depicts the overlay of the C-terminal Ca^2+^ of *Rf*DocScaA (purple) with the C-terminal Ca^2+^ of the group 4 dockerin of *Rf*DocCttA (cyan), whose coordination is also disrupted by a 13-residue long insert (dark green), but maintains an octahedral geometry due to the contribution of 2 water molecules (Wat). The structure of *Rf*DocScaA is colored tan, and the structure of *Rf*DocCttA is colored blue.

*Rf*DocScaA comprises three *α*-helices, two of which (helix 1: Val-662 to Asp-677; helix 3: Lys-710 to Leu-723) are arranged in an antiparallel orientation forming a planar surface on the Doc that interacts with CohScaB5 (Fig. 2). These two helices comprise portions of the two classic Doc repeating segments, each containing a bound Ca^2+^ ion in loops located at opposite ends of the module. However, much like in *R. flavefaciens* CttA XDoc^27^, the second repeating segment consists of an atypical variation of the EF-hand motif due to a large insertion in the Ca^2+^ binding loop (Fig. 2). This is the most defining characteristic of this module and will be further discussed below. Connecting these two structural elements is yet another *α*-helix (helix 2) extending from Asp-682 to Asp-689. The overall tertiary structure, with the exception of the loop insertion, bears some similarities to enzyme-associated dockerins from *C. thermocellum* (PDB code 3P0D: Z-score of 6.9, rmsd of 1.24 Å and 25% sequence identity over 65 aligned residues; PDB code 2CCL: Z-score of 6.5, rmsd of 1.44 Å and 26% sequence identity over 61 aligned residues) and *R. flavefaciens* (PDB code 5M2O: Z-score of 7.6, rmsd of 1.31 Å and 27% sequence identity over 68 aligned residues). The Ca^2+^ coordination in the N-terminal segment follows the typical *n, n* + *2, n* + *4, n* + *6, n* + 11 plus a water molecule (at the *n* + 8 position) pattern. Thus, the Ca^2+^ ion located at the N-terminus is coordinated by the side chains of Asp-653, Asn-655, Asp-657 and Asp-664 (both the Oδ1 and Oδ2), the latter belonging to α-helix 1 (Fig. 3A). The pentagonal bipyramid geometry of the coordination is completed by the main-chain carbonyl of Asp-659 and one water molecule (*n* + 8, *via* Asn-661) (Fig. 3A).

In contrast, the pattern of Ca^2+^ coordination in the C-terminal repeat is displaced due to the 12-residue loop insertion between Pro-693 and Ser-704 (Fig. 3B). A Phe residue replaces the usual Asn/Asp at position *n* + 2 and provides a backbone carbonyl oxygen ligand. The Asn/Asp at position *n* + 4 and water at position *n* + 8 are absent (Fig. 3B). Therefore, the coordination follows an atypical *n, n* + *2, n* + *18 (*at the *n* + 6 position), *n* + 23 (at the *n* + 11 position), pattern with no water molecules involved. Thus, the C-terminal Ca^2+^ coordination adopts a tetrahedral configuration involving the side chains of residues Asp-689 and Asp-712 (both the Oδ1 and Oδ2) and completed by the main-chain carbonyl groups of Phe-691 and Asp-707 (Fig. 3C). A similar atypical Ca^2+^-binding loop disruption has been observed in the *R. flavefaciens Rf*XDocCttA structure in complex with *Rf*CohScaE, where a 13-residue long insertion in the C-terminal loop also alters the Ca^2+^-coordination pattern in the Doc of the CttA protein, although the octahedral geometry is maintained thanks to the contribution of two water molecules (Fig. 3C)^27^. In *Rf*XDocCttA, it was found that the loop insert, together with two other inserts, serve as structural buttresses stabilizing the X-Module-Doc relationship. However, there is no X-module dyad associated with *Rf*DocScaA and therefore the function of the 12-residue flap remains unknown. Although the *Rf*DocScaA loop insert and the *Rf*XDocCttA insert have a similar location in the Doc structure, the fact they do not display significant sequence identity would appear to negate a direct evolutionary relationship.

A recent study suggested the existence of an intramolecular clasp between the N-terminal and C-terminal ends of DocScaA, which contributed to enhancing the stability of the Doc module^30^. Based on an *in silico* model of DocScaA from *R. flavefaciens* strain 17, the authors predicted a stacking interaction between an N-terminal tryptophan and a C-terminal proline^30^. By mutating those two residues a reduction in thermal and chemical stability was observed for the Doc^30^. The X-ray crystal structure of *Rf*DocScaA, observed here in complex with *Rf*CohScaB5, revealed the same stacking interaction between Trp-651 and Pro-727 (Figure S1), thus supporting the involvement of this crucial contact to maintain Doc structural integrity. Furthermore, these types of aromatic interactions are commonly involved in structural stabilization and similar intramolecular clasps have been identified in other known Docs^31–33^. Additional intramolecular contacts established by both ends of the protein module, such as the hydrogen bonds between Cys-690 and Asp-712/Ala-728 and between Asn-687 and Val-650/Gly-652 also provide additional structural stabilization to *Rf*DocScaA and contribute to its compact and globular conformation.

### *Rf*CohScaB5-DocScaA complex interface

**Figure 4:**
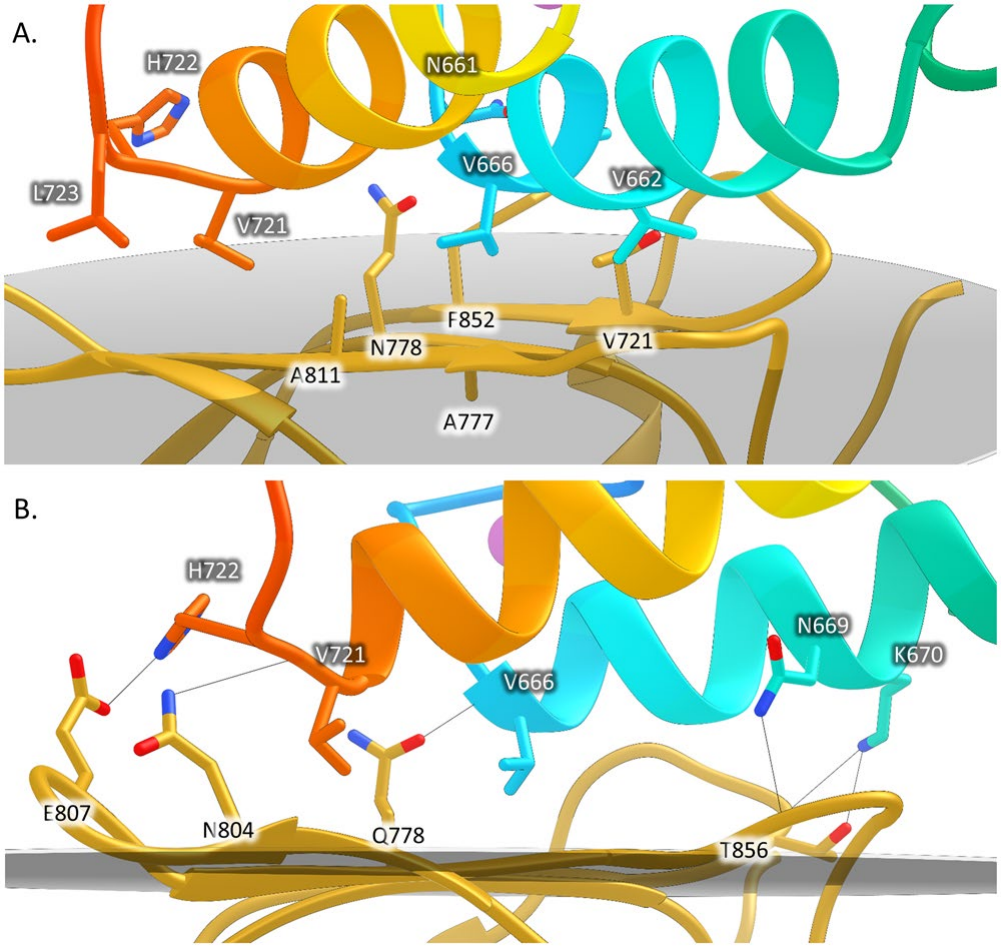
Cohesin-dockerin interface of *Rf*CohScaB5-DocScaA. Structure of *Rf*CohScaB5-DocScaA complex with a detailed view of the Coh-Doc interface showing the main hydrophobic contacts (Panel A) and main polar interactions (Panel B). In both panels the most important residues involved in Coh-Doc recognition are depicted in stick configuration, with a dark background label for the Doc residues and a light background label for the Coh residues, using the DocScaA and CohScaB5 numbering. Solid black lines mark hydrogen-bond interactions. Ca^2+^ ions are depicted as purple spheres. In all panels, the transparent grey disk marks the plane defined by the 8–3–6–5 β-sheet, where the β-strands form a distinctive dockerin-interacting plateau.

Helices 1 and 3 of *Rf*DocScaA make numerous contacts with *Rf*CohScaB5 *β*-sheets 8–3–6–5 (Fig. 4A,B). The Coh-interacting surface displays a flat topology although the loop connecting β-strands 8 and 9 is elevated from the plane defined by strands 8–3–6–5, thus leading the Coh into a closer proximity to the N-terminus of *Rf*DocScaA helix-1. Similarly the loop connecting β-strands 6 and 7 is also elevated in relation to the Coh plane promoting its interaction with the middle to the C-terminal portion of helix-1. This results in the entire length of *Rf*DocScaA helix-1 interacting with the Coh surface. In contrast, helix-3 interacts with the Coh platform predominantly through the C-terminus. Thus, *Rf*DocScaA displays a similar mechanism of Coh recognition to Group 1 Docs that also bind to ScaA or ScaB Cohs, predominantly through a single helix^28^. In contrast, *R. flavefaciens* group 3 and group 6 Docs interact with their Coh partners through the entire length of their two helices as previously observed in the *R. flavefaciens Rf*CohScaC-Doc3 complex. Thus, the two Doc3 *α*-helices (helix 1 and helix 3) of *Rf*CohScaC-Doc3 fully interact with CohScaC^22^ (Figure S2). A large network of polar (Table 2) and non-polar interactions (Table S1) stabilized the *Rf*CohScaB5-DocScaA complex interface (Fig. 4A,B). The interactions between the *α*-helix 1 of *Rf*DocScaA and the Coh are dominated by Doc residues Val-662, Ala-663, Val-666, Leu-667, Asn-669 and Lys-670 while the main contacting residues of Doc *α*-helix 3 include Ile-717, Val-720, Val-721, His-722 and Leu-723. The side chains of Val-662 and Val-666 at positions 11 and 15 dominate the hydrophobic recognition by contacting with the *Rf*CohScaB5 hydrophobic platform formed by Ala-775/777 and Phe812/852 (Fig. 4A). The significant hydrophobic contribution of the *α*-helix 1 to the interaction is reinforced by the contacts involving Ala-663 and the aliphatic regions of Lys-670, Asn-673, Asn-661 and Asn-669 of *Rf*DocScaA. The hydrogen bond network established by *α*-helix 1 is dominated by the interaction of Asn-669 with Glu-814 of *Rf*CohScaB5 and Lys-670 with Thr-856 (both Oγ1 and Oδ1) and Asn-857 of *Rf*CohScaB5 (Fig. 4B). An extra hydrogen bond is established between *Rf*DocScaA Val-666 main-chain N and *Rf*CohScaB5 Gln-778. In *α*-helix-3 the contacts are dominated by hydrophobic interactions involving Val-721, whose sidechain is positioned in the hydrophobic pocket created by Ala-811, Tyr-809, Tyr-810 and the aliphatic region of Asn-804 of *Rf*CohScaB5. Lys-710, Ile-717, Val-720, His-722 and Leu-723 reinforce the hydrophobic contacts of *α*-helix-3. The close proximity of the C-terminal portion of *α*-helix-3 also allows the establishment of an important hydrogen bond between Val-721 of *Rf*DocScaA and Asn-804 of *Rf*CohScaB5. In addition, a salt bridge is established between the Nδ1 atom of *Rf*DocScaA His-722 and the Oε1 atom of *Rf*CohScaB5 Glu-807.

**Table 2:** Main polar contacts between *Rf*CohScaB5 and *Rf*DocScaA.

	DocScaA				CohScaB5		
	Atom	Residue	Residue#		Atom	Residue	Residue #
**Hydrogen Bonds**							
H1	N	VAL	662	<>	OE1	GLN	778
H1	ND2	ASN	669	<>	O	GLU	814
H1	NZ	LYS	670	<>	O	THR	856
H1	NZ	LYS	670	<>	OG1	THR	856
H1	NZ	LYS	670	<>	OD1	ASN	857
H3	O	VAL	721	<>	ND2	ASN	804
**Salt Bridges**							
H3	ND1	HIS	722	<>	OE1	GLU	807

The table was made using the PDBePISA server. Dockerin residues are marked as belonging either to helix 1 (H1) or to helix 3 (H3) interfaces.

**Figure 5:**
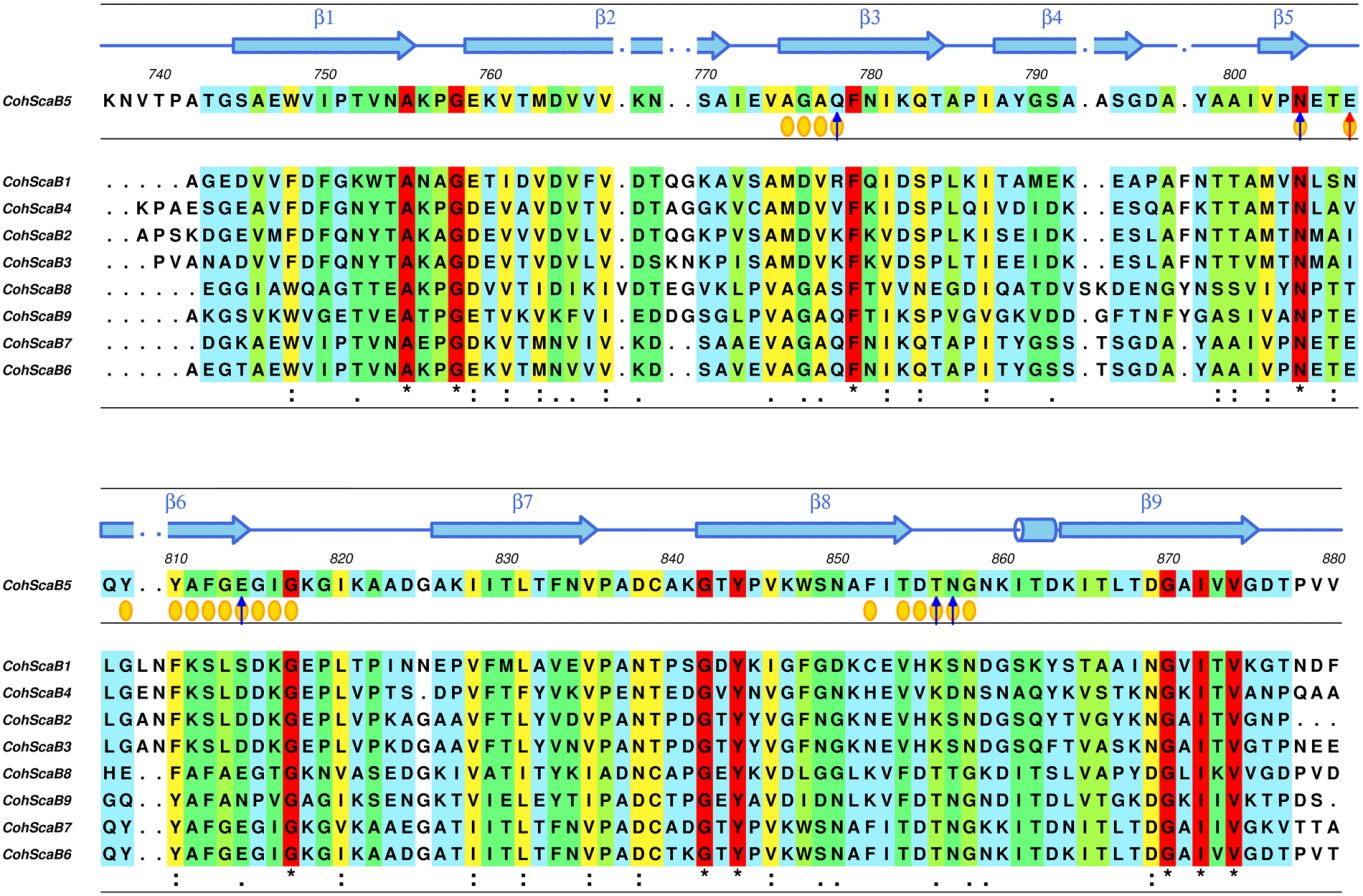
Multiple sequence alignment of *R. flavefaciens* ScaB cohesins 5 to 9. The primary sequence background is colored according to the ALSCRIPT Calcons convention, implemented in ALINE^51^: red, identical residues; orange to blue, lowering color-ramped scale of conservation. Above the alignment lies a cartoon representation of the secondary structure of *Rf*CohScaB5 (blue color) (Coh-Doc complex PDB codes: 5N5P). Residues involved in molecular interactions with the Doc partner are represented as follows: blue arrow for hydrogen bonds, red arrow for salt bridges and yellow circles for hydrophobic contacts.

The structure of the *Rf*CohScaB5-DocScaA complex revealed the residues of *Rf*CohScaB5 that recognize DocScaA. Previous work revealed that ScaB cohesins 5 to 9 display a similar binding specificity as these Cohs bind exclusively to the singular group 5 *Rf*ScaA Doc^21^. Alignment of the primary sequences of Cohs 5 to 9 (Fig. 5) provided a rationale for the conservation in binding specificity observed in these five Cohs. Thus, CohScaB5 residues Gln-778, Asn-804, Glu-807 and Thr-856, whose sidechains establish the main hydrogen bonds with DocScaA, are conserved in ScaB Cohs 6, 7 and 9. Interestingly, CohScaB8 Gln-778 and Glu-807 are replaced by hydroxy amino acids (Fig. 5). Whether these differences correspond to a lower affinity for DocScaA remains to be explored. CohScaB5 Ala-811 is also conserved in CohScaB6 to 9. Ala-811 lies in the hydrophobic pocket that accommodates the sidechains of DocScaA Val-662 and Val-721. In CohScaB1–4, this Ala is replaced by a Lys that will not allow these hydrophobic contacts and very likely result in steric clash with CohScaA. Thus, Ala-Lys replacement is an important determinant of Coh-Doc specificity within the *R. flavefaciens* cellulosome.

*Rf*DocScaA displays an exclusive binding specificity, as it is the only Doc that is able to recognize ScaB cohesins 5 to 9. The alignment of *Rf*DocScaA with the Doc sequences of ScaA scaffoldins recently discovered in diverse *R. flavefaciens* strains^17^ revealed the degree of conservation of residues involved in the recognition of ScaB Coh (Figure S3). Thus, within the 5 ScaA Doc homologues analyzed, residues Asn-661, Val-666, Asn-669 and Val-721 are completely conserved and Val-662 is replaced by an Ile in 2 strains. This conservation reinforces the importance of these residues for DocScaA’s ability to recognize CohScaB5.

### *Rf*ScaA presents a single binding mode

**Figure 6:**
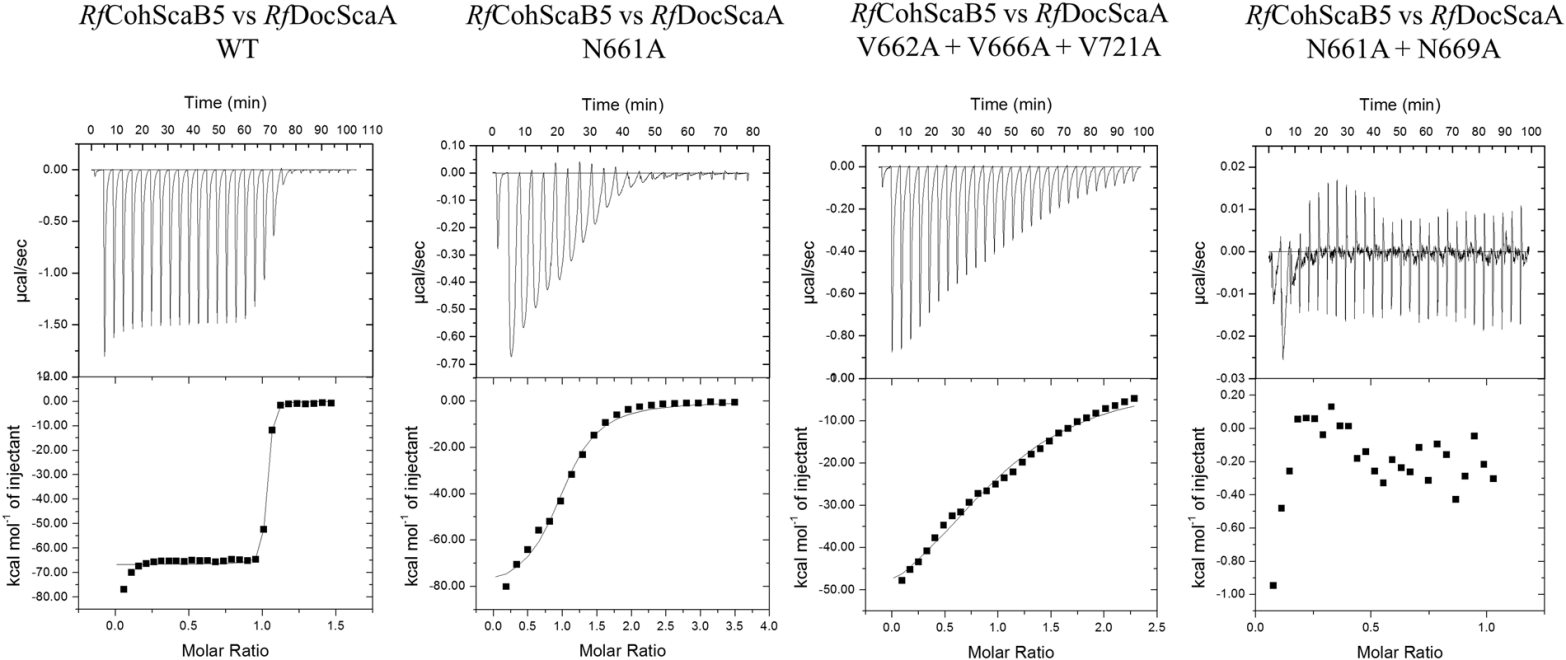
Binding affinity of CohScaB5 to DocScaA mutant derivatives and wild type partners as determined by ITC. Binding isotherms for: Panel A, *Rf*CohScaB5 vs *Rf*DocScaA; Panel B, *Rf*CohScaB5 vs *Rf*DocScaA N661A; Panel C, *Rf*CohScaB5 vs *Rf*DocScaA triple mutant; and Panel D, *Rf*CohScaB5 vs *Rf*DocScaA N661A + N669A double mutant. The upper part of each panel shows the raw heats of binding, whereas the lower parts comprise the integrated heats after correction for heat of dilution. The curve represents the best fit to a single-site binding model. The corresponding thermodynamic parameters are shown in Table 6.

Initially, non-denaturing gel electrophoresis (NGE) was used to probe the importance of *Rf*DocScaA residues for Coh recognition (Figure S4). The data revealed that single mutant derivatives of *Rf*DocScaA retain the capacity to interact with its protein partner, suggesting that the amino acid substitutions explored in this study had a marginal impact in affinity. Thus, to gain more insight into the driving forces of Coh-Doc recognition, the binding thermodynamics of *Rf*DocScA to *Rf*CohScaB5 were measured by isothermal titration calorimetry (ITC) at 308 K, which is the rumen approximate temperature of. The data (Table 3, Fig. 6), revealed a stoichiometry of 1:1 and a *K*_a_ of ~10^8^ M^−1^, similar to what was previously observed in other Coh-Doc interactions of *R. flavefaciens*^22,28^. However, an accurate determination of the *K*_a_ was not possible as affinity was close to the upper sensitivity range of the technique. The affinity of *Rf*DocScaA mutant derivatives described above for *Rf*CohScaB5 was also explored by ITC. An alanine substitution of *Rf*DocScaA residue Asn-661 resulted in a ~100-fold reduction in the affinity for *Rf*CohScaB5 (Table 3, Fig. 6). Even though the alanine substitutions of residues Val-662, Asn-669, Lys-670 and His-722 did not result in a decreased *K*_a_, the associated standard errors were lowered relative those observed for the wild-type interaction, which may indicate a reduction in affinity. The low impact that the alanine substitutions had on the affinity of the *Rf*CohScaB5-DocScaA interaction may reflect the inherent hydrophobic nature of the alanine sidechain and its ability to significantly compensate for the substitution. Overall, single mutations of DocScaA contacting residues seem to have little to no effect on the affinity to the Coh partner. However, combining any two of the tested valine mutations (Val-662, Val-666, Val-721) into *Rf*DocScaA double mutants resulted in a ~10-fold reduction in the affinity for *Rf*CohScaB5. Mutating all three *Rf*DocScaA valines led the *K*_*a*_ to decrease by approximately 1000 times relative to the estimated affinity of the wild type interaction (Table 3). The *Rf*DocScaA Asn-661Ala/Asn-669Ala double mutant derivative completely lost its capacity for *Rf*ScaBCoh5 recognition (Table 3). These data suggest that both polar and hydrophobic interactions play an important role in stabilizing the *Rf*CohScaB5-DocScaA interaction, with particularly relevant contributions provided by Val-662, Val-666 and Val-721.

**Table 3:** Thermodynamics of interaction between wild type CohScaB5 and wild-type and mutant variants of ScaDocA.

*Dockerin*	*K*_*a*_ *M* ^−1^	***Δ*** *G* ^*o*^ *kcal mol* ^−1^	***Δ*** *H kcal mol* ^−1^	−*T****Δ****S*^*o*^ *kcal mol*^−1^	*N*
DocScaA WT	4.02E8 ± 1.69E8	−12.14	−66.66 ± 0.585	54.51	1.01
DocScaA N661A	2.64E6 ± 4.49E5	−9.15	−82.45 ± 2.800	73.30	0.98
DocScaA V662A	4.78E8 ± 5.03E7	−12.32	−68.38 ± 0.110	56.05	1.02
DocScaA V666A	4.07E8 ± 1.86E8	−12.20	−56.56 ± 0.391	44.35	1.02
DocScaA N669A	3.16E8 ± 6.59E7	−12.12	−73.42 ± 0.322	61.29	1.01
DocScaA K670A	3.67E8 ± 4.33E7	−12.25	−75.09 ± 0.184	62.83	1.02
DocScaA V721A	5.10E8 ± 1.54E8	−12.35	−48.70 ± 0.199	36.34	0.95
DocScaA H722A	2.73E8 ± 7.38E7	−12.07	−72.44 ± 0.443	60.36	1.00
DocScaA N661 + N669	*Nb*	*Nb*	*Nb*	*Nb*	*Nb*
DocScaA V662 + V666	1.98E7 ± 1.44E6	−10.19	−67.48 ± 0.281	57.29	0.98
DocScaA V662 + V721	6.24E7 ± 9.36E6	−11.05	−64.33 ± 0.455	53.28	1.08
DocScaA V666 + V721	2.25E7 ± 1.58E6	−10.33	−56.53 ± 0.238	46.20	1.01
DocScaA V662 + V666 + V721	2.91E5 ± 3.79E4	−7.83	−64.81 ± 3.59	56.98	1.04

All thermodynamic parameters were determined at 308 K.

*Nb*, no binding.

A close inspection of the *Rf*CohScaB5-DocScaA complex structure suggests that *Rf*DocScaA residue Asn-661 does not play a critical role in *Rf*CohScaB5 recognition when compared with other residues, such as Val-662 and Val-666. However, Asn-661 is critically involved in the coordination of the N-terminal Ca^2+^, which may explain the decreased affinity observed when substituted with Ala. The methyl side-chain of Ala is unable to contribute to Ca^2+^ coordination, which is critical for maintaining the Doc fold and would thus negatively impact the interaction between the two modules. The thermogram resulting from the interaction between *Rf*ScaBCoh5 and the Asn-661Ala *Rf*DocScaA mutant is displayed in Fig. 6. Interestingly, the signals in the binding isotherm of this interaction appear to be broader suggestive of difference in kinetics of the interaction with *Rf*ScaBCoh5 compared to the wild-type *Rf*DocScaA. Thus, the decreased affinity revealed by *Rf*DocScaA single and multiple mutant derivatives where Asn-661 was replaced by an alanine may reflect an improper Doc fold rather than the importance of the residue to Coh recognition.

**Figure 7:**
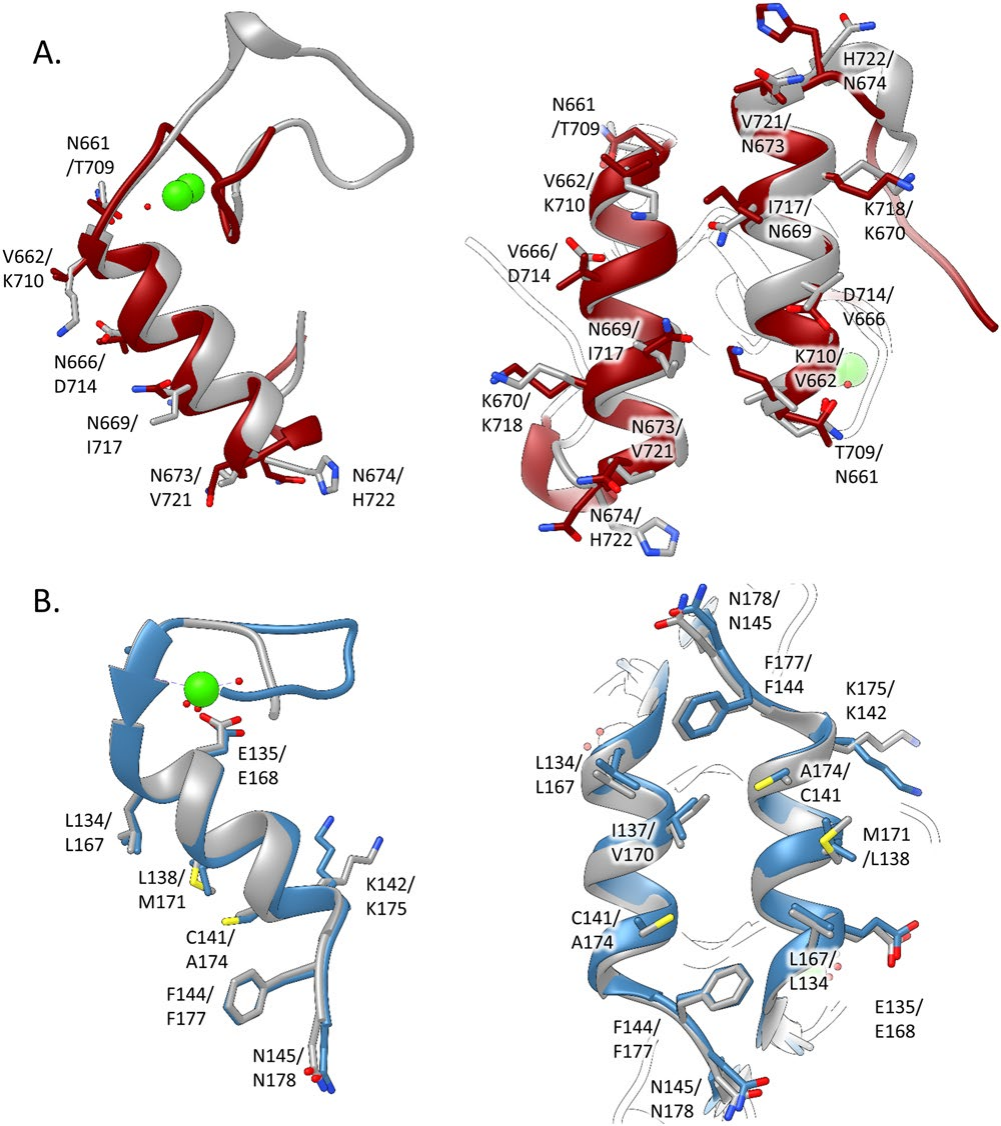
Non-symmetric and symmetric nature of Docs as exemplified by the structures of single-binding mode *Rf*DocScaA and dual-binding mode *Ac*DocScaA. Panel A, *R. flavefaciens* group5 Doc (DocScaA). Panel B, *A. cellulolyticus* DocScaA (*Ac*DocScaA). The left image of each panel shows an overlay of the N-terminal and C-terminal dockerin repeats. In both cases it is apparent that the 2 repeats are similar at the main-chain atoms but only the *Ac*DocScaA (Panel B) shows conservation in the side chains, allowing the dual-binding mode. The right image of each panel shows a comparison of the two putative binding surfaces by overlaying the dockerins with a version of themselves rotated by 180° (in grey), showing a lack of conservation in the key contacting residues in the *R. flavefaciens* dockerins (Panel A). Contrary to the *Ac*DocScaA (Panel B), lack of internal symmetry in *Rf*DocScaA and the involvement of both α1 and α3 helices in cohesin recognition suggest that they display a single cohesin-binding platform.

The observation that the Asn-661/Asn-669 mutant did not bind to *Rf*CohScaB5 suggests that *Rf*DocScaA presents a single-binding mode; although Asn-661 substitution affected Ca^2+^ coordination. It is plausible that under these conditions the symmetry-related helix-3 in the Doc structure could replace helix-1 supporting the recognition of *Rf*ScaBCoh5 through a symmetry-related interface. When Doc mudules present a dual-binding mode, mutation of a single or two residues positioned in the same helix usually has no effect on affinity, as a symmetry-related functional binding site can assume Coh recognition involving a 180° rotation of the Doc when binding its protein partner^34^. In addition, it has proven difficult to crystallize dual-binding mode complexes as these types of interactions present conformational heterogeneity that precludes crystal formation. Thus, this initial observation strongly suggested that *Rf*DocScaA presents a single-binding mode. To analyze the nature of structural symmetry observed within *Rf*DocScaA, the structure of *Rf*DocScaA was overlaid with itself after rotation of 180° in the Coh plane (Fig. 7A). The overlay suggests that residues Asn-661 and Asn-669 are replaced by Thr-709 and Ile-717, respectively, when the Doc is rotated by 180°, suggesting a disruption of the capacity of *Rf*DocScaA to recognize the Coh at these positions (Fig. 7A). Furthermore, the symmetry-related residues for valines 662, 666 and 721 are all of polar nature and therefore do not allow establishment of the extensive hydrophobic platform created by this critical valine triad. Overall these observations suggest that the asymmetric nature of *Rf*DocScaA leads to a unique mode for the formation of *Rf*CohScaB5-DocScaA complex. This contrasts with a large majority of Coh-Doc complexes where a dual-binding mode is observed, including those involving the binding of primary to adaptor scaffoldins as is the case for *Rf*CohScaB5-DocScaA. Thus, the symmetrical nature of *Acetivibrio cellulolyticus* DocScaA, which was previously shown to display a dual-binding mode by binding to cohesin *Ac*CohScaB3 in two distinct orientations (43), is easily demonstrated when its structure is overlaid with itself after a 180° rotation (Fig. 7B).

## Conclusions

The assembly of enzyme subunits into the *R. flavefaciens* strain FD-1cellulosome involves groups 1, 3 and 6 enzyme-borne Docs. Groups 3 and 6 Docs present essentially the same specificity, although a reversed binding mode, and recruit primarily hemicellulases to the multi-enzyme complex through the binding to the Coh of the *Rf*ScaC adaptor scaffoldin. *Rf*ScaC contains a group 1 Doc that, like the remaining 95 group 1 Docs, specifically binds Cohs of primary scaffoldin *Rf*ScaA as well as Coh 1 to 4 of adaptor scaffoldin *Rf*ScaB. Thus, group 1 Docs represent the major group of Docs, which recruit the largest number of enzymes to ruminal cellulosomes. Previous studies have revealed that group 1, 3 and 6 Docs essentially display a single-binding mode mechanism^22,28^. This contrasts with previous observations on the cellulosomes of *C. thermocellum*^34^, *A. cellulolyticus*^29^ and *C. cellulolyticum*^35^, in which Docs used to assemble the microbial enzymes into cellulosomes display a dual-binding mode. The 2-fold internal symmetry of dual-binding mode Docs permits the binding to the Coh partner in two 180°-related alternate positions. The observation that dual binding mode is highly conserved in bacterial Docs suggests that it might contribute to enhance the flexibility and accessibility in highly populated cellulosomes. Here, we have elucidated the structure of the unique *Rf*DocScaA in complex with the fifth Coh of *Rf*ScaB. The data revealed that, like groups 1, 3 and 6 Docs, the key *Rf*DocScaA lacks the internal symmetry previously observed in most cellulosomal Docs. Thus, the recent work in *R. flavefaciens* strain FD-1cellulosomal protein-protein interactions reveals that the dual-binding mode is not universal to all cellulosomal systems and suggests that ruminal cellulosomes are assembled predominantly through single-binding mode Docs. This is a rather striking observation as the dual-binding mode was believed to universally improve the flexibility of highly populated cellulosomal systems. While it is possible that the dual-binding mode allows Docs, in cellulosomes with a limited scaffoldin repertoire, to explore a larger space by having alternate conformations, it is also possible that the dual-binding mode represents an adaptation to the physicochemical properties of different ecological niches. The fact that CAZymes have spread through bacteria and fungi essentially through horizontal gene transfer, suggests that the same mechanism operated to exchange the other components of cellulosomal systems^36,37^. As such, it is probable that all Docs evolved from a common ancestral sequence through a mechanism involving gene duplication and subsequent horizontal gene transfer. Thus, it is likely that the dual-binding mode is a property maintained from ancestral Docs, and it should confer a competitive selective advantage in the majority of the cellulosome systems described to date. The biochemical factors that constitute the driving selective force for the evolution of single-binding mode Docs remain to be elucidated. It is possible that the highly stable physical-chemistry properties revealed by the rumen do not require highly flexible Coh-Doc interactions for efficient cellulosome assembly and function but this hypothesis remains to be tested.

## Methods

### Gene synthesis and DNA cloning

Docs are highly unstable when produced recombinantly in *Escherichia coli*. To promote stability, *R. flavefaciens* FD-1 DocScaA (WP_009986657.1 residues 648–730) was co-expressed *in vivo* with CohScaB5 (WP_009986658.1 residues 737–880). The immediate binding of DocScaA to CohScaB5 is believed to confer immediate stabilization of the Doc structure. The genes encoding the two proteins were designed to maximize expression in *E. coli*, synthesized *in vitro* (NZYTech Portugal) and cloned into pET28a (Merck Millipore, Germany) under the control of separate T7 promoters. The DocScaA-encoding gene was at the 5′ end while the CohScaB5-encoding gene was at the 3′ end of the synthetic DNA. A T7 terminator sequence (to terminate transcription of the dockerin gene) and a T7 promoter sequence (to control transcription of the cohesin gene) were incorporated between the sequences of the two genes. *Nhe*I and *Nco*I recognition sites at the 5′ end and *Xho*I and *Sal*I at the 3′ end were specifically inserted to allow subcloning into pET28a (Merck Millipore, Germany), such that the sequence encoding a six-residue His tag could be introduced either at the N-terminus of the dockerin (through digestion with *Nhe*I and *Sal*I, incorporating the additional sequence MGSSHHHHHHSSGLVPRGSHMAS N-terminal of the Doc) or at the C-terminus of the cohesin (by cutting with *Nco*I and *Xho*I, which incorporates the additional sequence LEHHHHHH C-terminal of the Coh). Thus, the two pET28a plasmid derivatives led to the expression of protein complexes with the engineered hexa-histidine either located at the dockerin or the cohesin. The two separate plasmids were used to express *Rf*CohScaB5-DocScaA complexes in *E. coli*. The sequences of DocScaA and CohScaB5 are presented in Table S2.

Recombinant cohesins and dockerins were produced individually by using two distinct cloning strategies. First, to express the cohesin individually the previously described cohesin-tagged version of the pET28 derivative was digested with *Bgl*II to remove the dockerin sequence. This strategy gave a pET28a derivative encoding the recombinant cohesin CohScaB5 fused to a C-terminal hexa-histidine tag. The DocScaA-encoding gene was cloned into the pHTP2 vector (NZYtech, Lisbon, Portugal) following the manufacturer’s protocol. Dockerin genes were isolated from *R. flavefaciens* FD-1 genomic DNA by PCR and using the primers shown in Table S3. The recombinant dockerin encoded by the pHTP2 derivatives contained an N-terminal thioredoxin A and an internal hexa-histidine tag for increased protein stability and solubility. Sequences of all plasmids produced were verified by Sanger sequencing.

To identify the Doc residues that modulate Coh recognition, several TrxADocScaA protein derivatives were produced using site directed mutagenesis. PCR amplification of the Doc-containing plasmid, using the primers presented in Table S3, allowed the production of seven DocScaA protein derivatives, namely N661A, V662A, V666A, N669A, K670A, V721A, H722A. Each of the newly generated gene sequences was fully sequenced to confirm that only the desired mutation accumulated in the nucleic acid.

### Expression and purification of recombinant proteins

Initial expression studies revealed that when the polyhistidine tag was located at the Doc N-terminal end in *Rf*CohScaB5-DocScaA complexes, the expression levels of both Coh and Doc were elevated. Expressing the cohesin with the histidine tag led to the accumulation of of unbound cohesin, which suggests that either the cohesin expresses at higher levels than the dockerin or that the untagged dockerin was less stable. Therefore, the construct encoding the protein complex with the tagged dockerin was subsequently selected to upscale the production of the *Rf*CohScaB5-DocScaA complex. BL21 (DE3) *E. coli* cells were transformed with the vector containing the construct and grown at 37 °C to an OD_600_ of 0.5. 1 mM isopropyl *β*-D-1-thiogalactopyranoside was added to induce recombinant protein expression, followed by incubation at 19 °C for 16 hours. After harvesting the cells by centrifuging 15 min. at 5000 × *g*, the cells were resuspended in 20 mL of immobilized-metal affinity chromatography (IMAC) binding buffer (50 mM HEPES, pH 7.5, 10 mM imidazole, 1 M NaCl, 5 mM CaCl_2_). Disruption of the cells was done through sonication and the cell-free supernatant was then recovered by centrifuging 30 min. at 15,000 × *g*. After loading the soluble fraction into a HisTrap^TM^ nickel-charged Sepharose column (GE Healthcare, UK), initial purification was carried out by IMAC in a FPLC system (GE Healthcare, UK) using conventional protocols with a 35 mM imidazole wash and a 35–300 mM imidazole elution gradient. After selecting the fractions containing the cohesin–dockerin complex, the buffer of the purified samples was changed to 50 mM HEPES, pH 7.5, containing 200 mM NaCl, 5 mM CaCl_2_ using a PD-10 Sephadex G-25M gel-filtration column (Amersham Pharmacia Biosciences, UK). Gel-filtration chromatography using a HiLoad 16/60 Superdex 75 cloumn (GE Healthcare, UK) was used as a second purification step. The purified complex samples were concentrated in an Amicon Ultra-15 centrifugal device with a 10-kDa cutoff membrane (Millipore, USA) and washed three times with molecular biology grade water (Sigma) containing 0.5 mM CaCl_2_. The final protein concentration was adjusted to 45 mg mL^−1^. Protein concentration was estimated in a NanoDrop 2000c spectrophotometer (Thermo Scientific, USA) using a molar extinction coefficient (ε) of 31 065 M^−1^ cm^−1^. The storage buffer consisted in molecular biology grade water containing 0.5 mM CaCl_2_. 14% (w/v) SDS–PAGE gels were used to confirm the purity and molecular mass of the recombinant complexes.

His GraviTrap gravity-flow nickel-charged Sepharose columns (GE Healthcare, UK) were used to purify the TrxADocScaA mutant derivatives and CohScaB5 used in native PAGE and ITC experiments.

### Nondenaturing gel electrophoresis (NGE)

For the NGE experiments, the proteins were kept in the IMAC elution buffer (50 mM HEPES, pH 7.5, 300 mM imidazole, 1 M NaCl, 5 mM CaCl_2_). Each of the TrxADocScaA variants, at a concentration of 15 μM, was incubated in the presence and absence of 15 μM CohScaB5 for 30 min at room temperature and separated on a 10% native polyacrylamide gel. Electrophoresis was carried out at room temperature. The gels were stained with Coomassie Blue. Complex formation was detected by the presence of an additional band displaying a lower electrophoretic mobility than the individual modules.

### Isothermal titration calorimetry

All ITC experiments were carried out at 308 K. The buffer used consisted in 50 mM HEPES pH 7.5, 0.5 mM CaCl_2_ and 0.5 mM TCEP. The purified TrxADocScaA variants and CohScaB5 were diluted to the required concentrations and filtered using a 0.45-μm syringe filter (PALL). Protein concentration was estimated in a NanoDrop 2000c spectrophotometer (Thermo Scientific, USA) using a molar extinction coefficient (ε) of 31 065 M^−1^ cm^−1^. During titrations, the Doc constructs were stirred at 307 revolutions/min in the reaction cell and titrated with 28 successive 10 μL injections of CohScaB5 at 220-s intervals. Integrated heat effects, after correction for heats of dilution, were analyzed by nonlinear regression using a single-site model (Microcal ORIGIN version 7.0, Microcal Software, USA). The fitted data yielded the association constant (K_A_) and the enthalpy of binding (ΔH). Other thermodynamic parameters were calculated using the standard thermodynamic equation: *ΔRTlnK*_*A*_ = *ΔG* = *ΔH* − *TΔS*.

### X-ray crystallography, structural determination and refinement

Several crystallization conditions were tested by using the sitting-drop vapor-diffusion method with the aid of an Oryx8 robotic nanodrop dispensing system (Douglas Instruments, UK^38^). The commercial kits JCSG+ HT96 (Molecular Dimensions, UK), Crystal Screen, PEG/Ion (Hampton Research, California, USA), and an in-house screen (80 factorial) were used for the screening. 1 µl drops of 12.5, 25 and 45 mg ml^−1^ *Rf*CohScaB5-DocScaA were mixed with 1 µl reservoir solution at room temperature. The resulting plates were then stored at 292 K. Crystal formation was observed under 2 conditions (0.1 M HEPES pH 7.5, 1.2 M sodium citrate; 2.1 M DL-malic acid pH 7.0) after a period of approximately 180 days from setting up the plates (maximum dimensions ~50 × 50 × 20 μm). These crystals were cryoprotected with mother solution containing 20–30% glycerol and flash-cooled in liquid nitrogen. Preliminary X-ray diffraction experiments revealed that these crystals were of very poor quality mainly due to high mosaicity. Optimization plates based on the 2 original hits were set up. Two additive plates (one for each original condition) were also set up using the HT Additive Screen (Hampton Research, California, USA). The additive screen drops consisted of 0.8 µl protein +0.8 µl optimization condition +0.2 µl stock additive solution. This approach generated several good quality crystals. X-ray diffraction data were collected on beamline PROXIMA-1 at the Soleil Synchrotron, Saint-Aubin, France using a PILATUS 6M detector (Dectris Ltd) from crystals cooled to 100 K with a Cryostream (Oxford Cryosystems Ltd). A systematic grid search was carried out on all of these crystals to select the best diffracting part of each crystal. EDNA^39^ and iMosflm^40^ were used for strategy calculation during data collection. All data sets were processed using the Fast_dp and xia2^41^ packages, which use the programs XDS^42^, POINTLESS and SCALA^43^ from the CCP4 suite^44^. Data-collection statistics are given in Table 1.

The best diffracting crystal was formed in one of the additive screen conditions (0.1 M HEPES pH 7.5, 1.2 M Sodium Citrate, 4% v/v acetonitrile). It diffracted to a resolution of 1.4 Å and belonged to the monoclinic spacegroup P2_1_. Phaser MR was used to carry out molecular replacement^45^. The best solution was found using a cohesin from *R. flavefaciens* strain 17 ScaB (unreleased) and an ensemble of 3 *R. flavefaciens* FD-1 dockerins (Doc1a from 5M2O, Doc1b from 5M2S and Doc3 from 5LXV) produced with Dali^46^. The cohesin had a sequence identity of 33.0% and the dockerins between 22% (Doc3) and 34% (Doc1b). Two copies of the heterodimer *Rf*CohScaB5-DocScaA complex were present in the asymmetric unit. The partially obtained model was completed with Buccaneer^47^ and with manual modeling in COOT. It was then refined using REFMAC5^48^ and PDB REDO^49^ interspersed with model adjustment in COOT. The final round of refinement was performed using the TLS/restrained refinement procedure using each module as a single group, giving the final model (Protein Data Bank code 5N5P, Table 1). The root mean square deviation of bond lengths, bond angles, torsion angles and other indicators were continuously monitored using validation tools in COOT and MOLPROBITY. A summary of the refinement statistics is provided in Table 1.

## Electronic supplementary material

Supplementary Information
