# Supplementary material for: Higher order scaffoldin assembly in Ruminococcus flavefaciens cellulosome is coordinated by a discrete cohesin-dockerin interaction
Source: Sci Rep. 2018 May 3;8:6987. doi: 10.1038/s41598-018-25171-8 (PMC5934362; doi:10.1038/s41598-018-25171-8)
Supplement: Supplementary file 1 — Supplementary Information [file 41598_2018_25171_MOESM1_ESM.pdf]

Higher order scaffoldin assembly in *Ruminococcus flavefaciens* cellulosome is coordinated by a discrete cohesin-dockerin interaction

Pedro Bule[a,*], Virgínia M.R. Pires[a], Victor D. Alves[a], Ana Luísa Carvalho[b], José A.M. Prates[a], Luís M.A. Ferreira[a], Steven P. Smith[c], Harry J. Gilbert[d], Ilit Noach[e], Edward A. Bayer[e], Shabir Najmudin[a] and Carlos M.G.A. Fontes[a,f,*]

Running title: Structure of *R. flavefaciens* Coh-Doc complex

[a] *CIISA – Faculdade de Medicina Veterinária, ULisboa, Pólo Universitário do Alto da Ajuda, Avenida da Universidade Técnica, 1300-477 Lisboa, Portugal*;
[b] *UCIBIO-REQUIMTE, Departamento de Química, Faculdade de Ciências e Tecnologia, Universidade Nova de Lisboa, 2829-516 Caparica, Portugal;*
[c] *Department of Biomedical and Molecular Sciences, Queen's University, Kingston, ON K7L 3N6, Canada*;
[d] *Institute for Cell and Molecular Biosciences, Newcastle University, The Medical School, Newcastle upon Tyne NE2 4HH, United Kingdom;*
[e] *Department of Biomolecular Sciences, The Weizmann Institute of Science, Rehovot 76100 Israel;*
[f] *NZYTech genes & enzymes, Estrada do Paço do Lumiar, 1649-038 Lisboa, Portugal.*

[*] Corresponding authors:
E-mail: pedrobuleg@gmail.com, cafontes@fmv.ulisboa.pt

Keywords: Cellulosome, protein-protein interaction, protein structure, rumen, cellulose, cellulase, scaffoldin protein.

Data deposition: Coordinates and observed structure factor amplitudes have been deposited in the Protein Data Bank with the wwPDB entry code 5N5P.

Table S1. Main hydrophobic contacts between *Rf*CohScaB5 and *Rf*DocScaA. Table was made using the PDBePISA server. Some of the dockerin residues are marked as belonging either to helix 1 (H1) or to helix 3 (H3) interfaces.

DocScaA					CohScaB5
		**Residue**	**Residue #**		**Residues**
		ASN	661	< >	GLN778 (5), PHE852 (7),
H1		VAL	662	< >	GLY776, ALA777 (4), GLN778 (5), PHE812, PHE852, THR854 (3)
H1		ALA	663	< >	THR854 (3), GLY858 (2)
H1		VAL	666	< >	ALA775 (4), GLY817 (3), THR854, ASP855, THR856
H1		LEU	667	< >	ASN857 (2)
H1		ASN	669	< >	GLU814 (4), GLY815 (7), ILE816 (6), GLY817
H1		LYS	670	< >	ILE816 (3), THR856 (5), ASN857 (4)
H1		ASN	673	< >	ILE816 (6)
	H3	LYS	710	< >	GLY815
	H3	ILE	717	< >	PHE812, GLY813
	H3	VAL	720	< >	GLN778 (3), TYR809 (3),
	H3	VAL	721	< >	ASN804 (3), TYR809 (6), TYR810, ALA811 (2)
	H3	HIS	722	< >	ASN804 (2), GLU807 (6), TYR809 (3)
	H3	LEU	723	< >	PRO803 (2), ASN804 (3)

Table S2. Recombinant protein sequences of *Rf*CohScaB5, *Rf*DocScaA and mutant variants of the latter produced for the interaction studies. The mutated residues are highlighted in black. The underline sequences correspond to the Dockerin's TrxA-His6x and the Cohesin's His6x tags.

Protein	Sequence
*Rf*DocScaA WT	MSDKIIHLTDDSFDTDVLKADGAILVDFWAEWCGPCKMIAPILDEIADEYQGKLTVAKLNIDQNPGTAPKYGIRGIPTLLLFKNGEVAATKV GALSKGQLKEFLDANLAGSGSGHMHHHHHHSSMTSLYKKAGFGNTLKPVWGDVNCDGDVNVADVVLLNKWLNNNADYAMTDQGKV NADCFNPQDANGGAVDASKVDLTKADSDAIIKSVVHLITLPAKG
*Rf*DocScaA N661A	AGFGNTLKPVWGDVNCDGDVAVADVVLLNKWLNNNADYAMTDQGKVNADCFNPQDANGGAVDASKVDLTKADSDAIIKSVVHLIT…
*Rf*DocScaA V662A	AGFGNTLKPVWGDVNCDGDVNAADVVLLNKWLNNNADYAMTDQGKVNADCFNPQDANGGAVDASKVDLTKADSDAIIKSVVHLIT…
*Rf*DocScaA V666A	AGFGNTLKPVWGDVNCDGDVNVADVALLNKWLNNNADYAMTDQGKVNADCFNPQDANGGAVDASKVDLTKADSDAIIKSVVHLIT…
*Rf*DocScaA N669A	AGFGNTLKPVWGDVNCDGDVNVADVVLLAKWLNNNADYAMTDQGKVNADCFNPQDANGGAVDASKVDLTKADSDAIIKSVVHLIT…
*Rf*DocScaA K670A	AGFGNTLKPVWGDVNCDGDVNVADVVLLNAWLNNNADYAMTDQGKVNADCFNPQDANGGAVDASKVDLTKADSDAIIKSVVHLIT…
*Rf*DocScaA V721A	AGFGNTLKPVWGDVNCDGDVNVADVVLLNKWLNNNADYAMTDQGKVNADCFNPQDANGGAVDASKVDLTKADSDAIIKSVAHLIT…
*Rf*DocScaA H722A	AGFGNTLKPVWGDVNCDGDVNVADVVLLNKWLNNNADYAMTDQGKVNADCFNPQDANGGAVDASKVDLTKADSDAIIKSVVALIT…
*Rf*DocScaA N661A + N669A	AGFGNTLKPVWGDVNCDGDVAVADVVLLAKWLNNNADYAMTDQGKVNADCFNPQDANGGAVDASKVDLTKADSDAIIKSVVHLIT…
*Rf*DocScaA V662A + V666A	AGFGNTLKPVWGDVNCDGDVNAADVALLNKWLNNNADYAMTDQGKVNADCFNPQDANGGAVDASKVDLTKADSDAIIKSVVHLIT…
*Rf*DocScaA V662A + V721A	AGFGNTLKPVWGDVNCDGDVNAADVVLLNKWLNNNADYAMTDQGKVNADCFNPQDANGGAVDASKVDLTKADSDAIIKSVAHLIT…
*Rf*DocScaA V666A + V721A	AGFGNTLKPVWGDVNCDGDVNVADVALLNKWLNNNADYAMTDQGKVNADCFNPQDANGGAVDASKVDLTKADSDAIIKSVAHLIT…
*Rf*DocScaA V662A + V666A + V721A	AGFGNTLKPVWGDVNCDGDVNAADVALLNKWLNNNADYAMTDQGKVNADCFNPQDANGGAVDASKVDLTKADSDAIIKSVAHLIT…
*Rf*CohScaB5 WT	MGSSHHHHHHSSGLVPRGSHMASKNVTPATGSAEWVIPTVNAKPGEKVTMDVVVKNSAIEVAGAQFNIKQTAPIAYGSAASGDAYAAIV PNETEQYYAFGEGIGKGIKAADGAKIITLTFNVPADCAKGTYPVKWSNAFITDTNGNKITDKITLTDGAIVVGDTPVV

Table S3. Set of primers used to isolate the *Rf*DocScaA gene and to generate its mutant derivatives. The fraction in capital letters corresponds to the homologous recombination zone. Mutated codons are shown bold and underlined.

ID	Vector	Primers used
***Rf*DocScaA**	pETG20A	5′ GGGGACAAGTTTGTACAAAAAAGCAGGCTTC cctgcagaaacaacaactacag<br>3′ GGGGACCACTTTGTACAAGAAAGCTGGGTC ttagcccttagcagggagtgtgatg
***Rf*DocScaA N661A**	pETG20A	5′ ctgcgacggtgacgta**gcc**gtagctgacgttgttc<br>3′ gaacaacgtcagctacggctacgtcaccgtcgcag
***Rf*DocScaA V662A**	pETG20A	5′ gacggtgacgtaaac**gca**gctgacgttgttctc<br>3′ gagaacaacgtcagctgcgtttacgtcaccgtC
***Rf*DocScaA V666A**	pETG20A	5′ gtaaacgtagctgacgtt**gct**ctccttaacaagtgg<br>3′ ccacttgttaaggagagcaacgtcagctacgtttac
***Rf*DocScaA N669A**	pETG20A	5′ gacgttgttctcctt**gcc**aagtggctcaacaac<br>3′ gttgttgagccacttggcaaggagaacaacgtc
***Rf*DocScaA K670A**	pETG20A	5′ gttgttctccttaac**gcg**tggctcaacaacaatg<br>3′ cattgttgttgagccacgcgttaaggagaacaac
***Rf*DocScaA V721A**	pETG20A	5′ ctatcatcaagagcgta**gct**cacctcatcacactc<br>3′ gagtgtgatgaggtgagctacgctcttgatgatag
***Rf*DocScaA H722A**	pETG20A	5′ catcaagagcgtagtt**gcc**ctcatcacactccctg<br>3′ cagggagtgtgatgagggcaactacgctcttgatg
***Rf*DocScaA V662A + V666A**	pETG20A	5′ gtgacgtaaac**gca**gctgacgtt**gct**ctccttaacaagtg<br>3′ cacttgttaaggagagcaacgtcagctgcgtttacgtcac

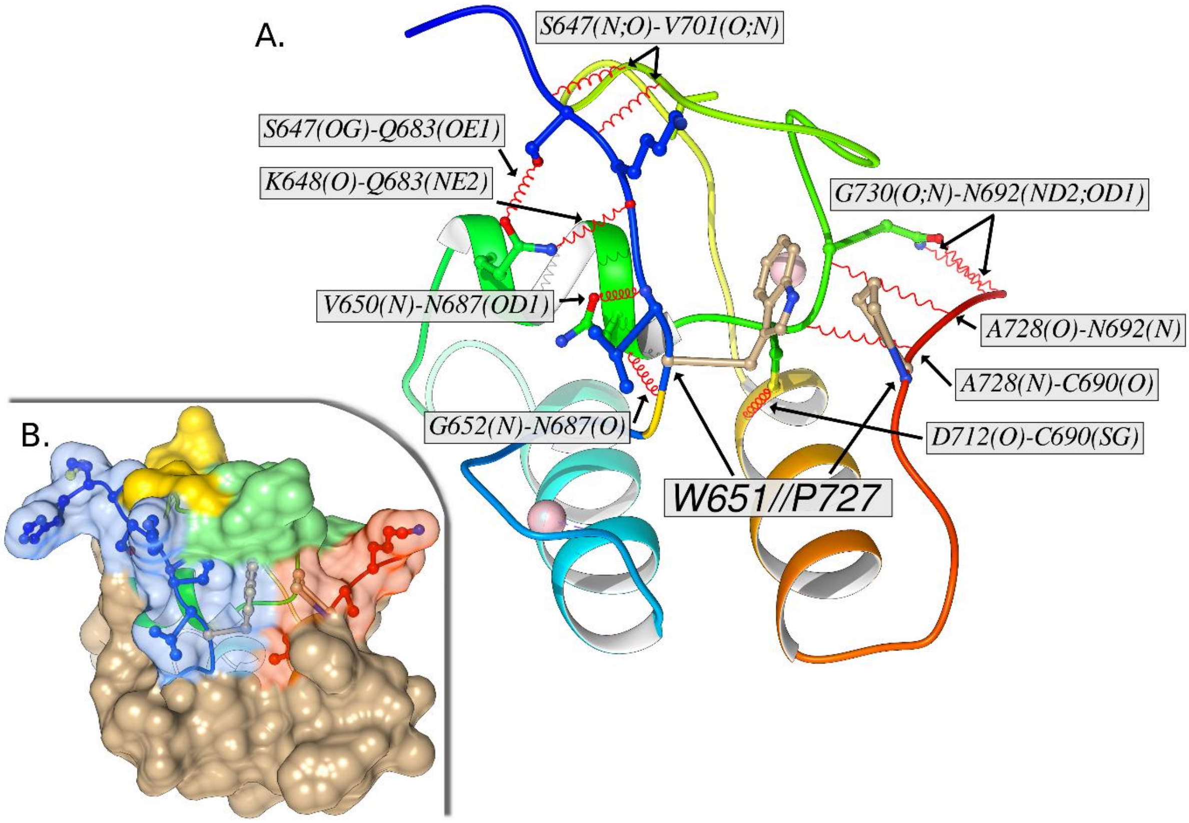

FIGURE S1. The most important intramolecular contacts for the stabilization of the dockerin module. Panel A shows the structure of *Rf*DocScaA represented in color ramped style from the blue N-terminus to the red C-terminus. The sidechains of residues Trp-651 and Pro-727, which make an important stacking interaction are represented as ball & stick and colored tan. The most important hydrogen bond contacts involved in structure stabilization are represented as red springs and the residues making those contacts have their sidechains highlighted in ball&stick representation. Gly-652 is highlighted in yellow due to the lack of a sidechain. In panel B the molecular surface *Rf*DocScaA is represented in tan and shows the dockerin globular conformation supported by the extensive network of intramolecular contacts established by two ends of the protein, both between themselves and with other regions. The N- and C-terminal regions are highlighted in blue and red, respectively.

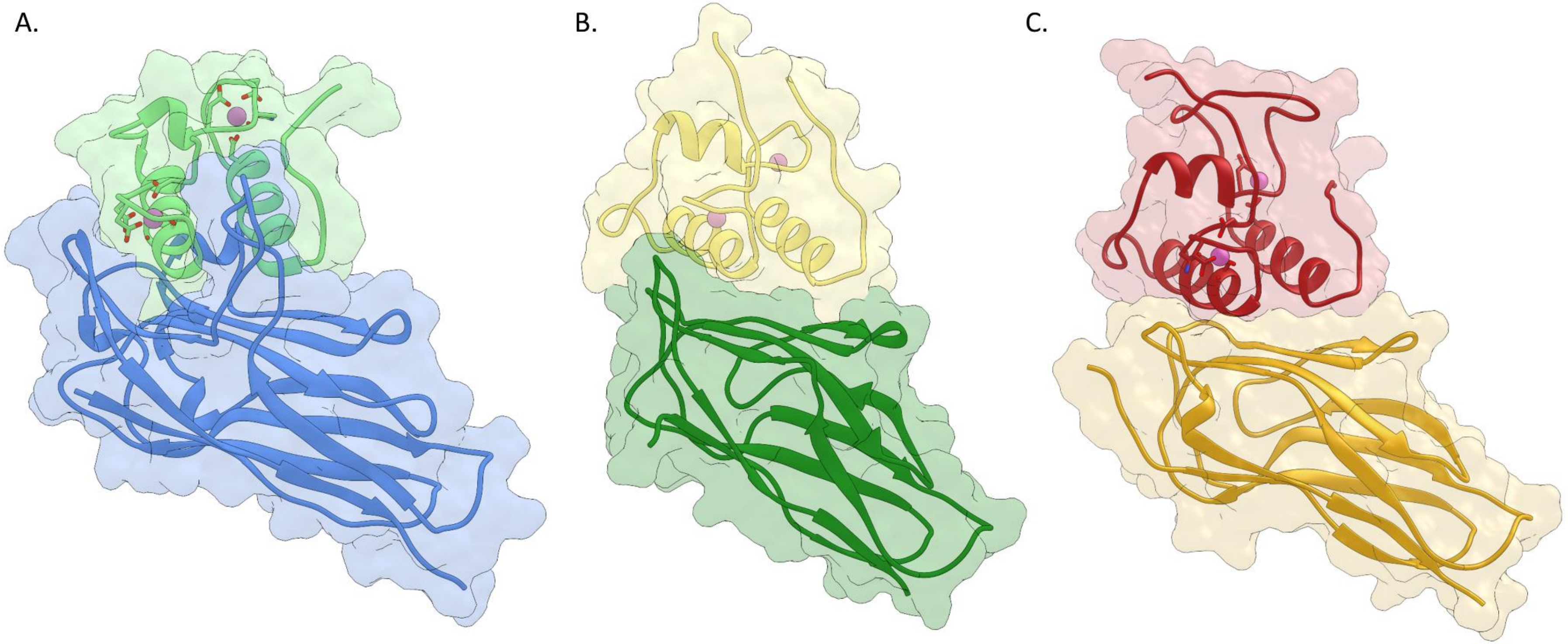

FIGURE S2. Structure of the three *R. flavefaciens* Coh-Doc complex specificities responsible for cellulosomal assembly. Panel A depicts the structure of *Rf*CohScaC-Doc3 with the dockerin in light green and the cohesin in blue. This complex is responsible for recruiting group 3 and 6 dockerin associated enzymes *via* the ScaC adaptor scaffoldin to *R. flavefaciens* cellulosome. Panel B displays the structure of *Rf*CohScaB3-Doc1a with the dockerin in light yellow and the cohesin in dark green. This interaction is responsible for the integration of group 1 dockerin associated proteins directly to primary scaffoldins ScaA and ScaB. Group 1 Docs are the major Doc group in *R. flavefaciens*. Panel C shows the structure of *Rf*CohScaB5-DocScaA with the dockerin in dark red and the cohesin in gold. This interaction is responsible for attaching up to 5 ScaA primary scaffoldins onto a single ScaB primary/adaptor scaffoldin.

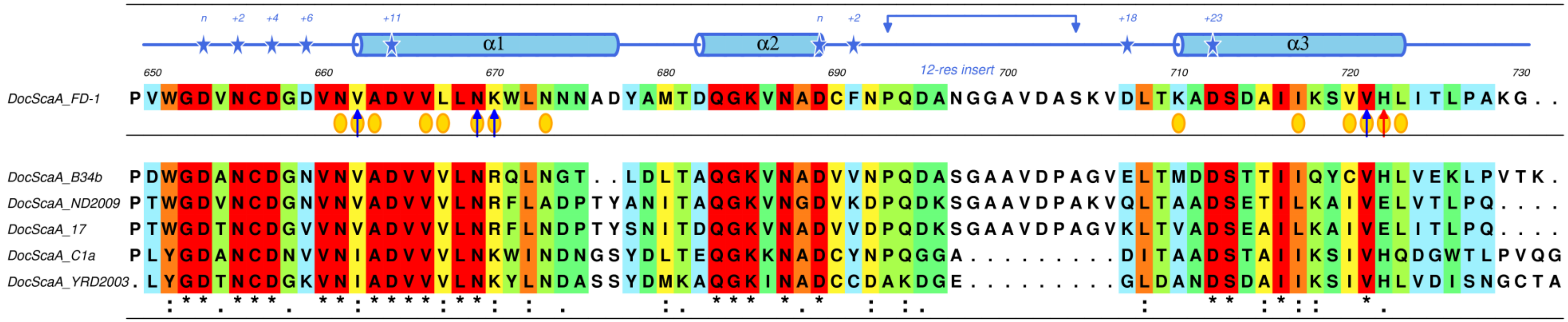

FIGURE S3. Multiple sequence alignment of *Rf*DocScaA with its closest primary structure homologues. The primary sequence background is colored according to the ALSCRIPT Calcons convention, implemented in ALINE (49): red, identical residues; orange to blue, lowering color-ramped scale of conservation. Above the alignment lies a cartoon representation of the secondary structure of *Rf*DocScaA from strain FD-1 (blue color) (Coh-Doc complex PDB code: 5N5P). Also, the residues involved in molecular interactions with the Coh partner are represented as follows: blue arrow for hydrogen bonds, red arrow for salt bridges and yellow circles for hydrophobic contacts.

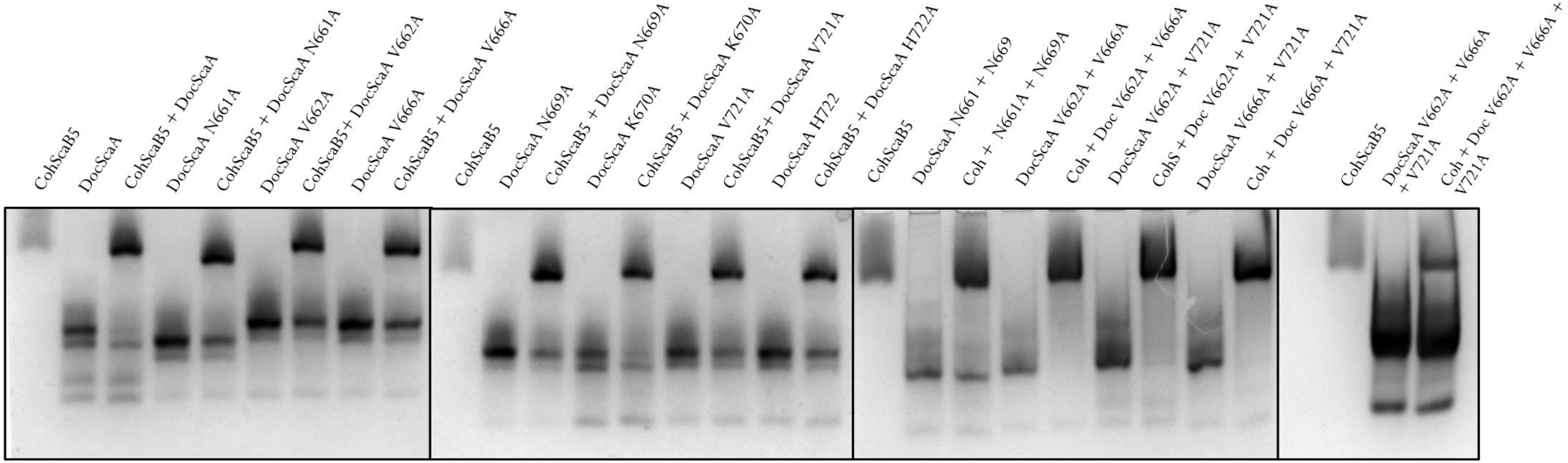

FIGURE S4. Binding affinity of CohScaB5 to DocScaA and its mutant derivatives as determined by NGE. In Panel A the lanes marked CohScaB5 were loaded with the Coh. Adjacent lanes were loaded with the dockerin mutant derivatives and with both Coh and Doc modules after 60-min incubation at equimolar concentrations. The appearance of a band with a different migration pattern in lanes containing the complex represents a positive result (e.g. CohScaB5 + DocScaA3), while a negative result (e.g. Coh + N661A + N669A) is given by the presence of only the individual dockerin and cohesin bands. The edges of the four non-denaturing polyacrylamide gels are defined by the black outline. The rightmost gel has been cropped since the remaining wells were not used.
